# SOFT-TISSUE COMPOSITION ASYMMETRY PREDICTS LEG MUSCLE STRENGTH CHANGES OVER FIVE YEARS AMONG ICELANDIC OLDER ADULTS

**DOI:** 10.1093/geroni/igae098.0812

**Published:** 2024-12-31

**Authors:** Milan Chang (Gudjonsson), Marco Recenti, Carlo Ricciardi, Alfonso Maria Ponsiglione, Francesco Amato, Magnus Kjartan Gislason, Paolo Gargiulo

**Affiliations:** Landspitali University Hospital, Seltjarnarnes, Hofuoborgarsvaoio, Iceland; Reykjavik University, Reykjavik, Hofuoborgarsvaoio, Iceland; University of Naples Federico II, Naples, Calabria, Italy; University of Naples Federico II, Naples, Campania, Italy; Università degli Studi Magna Græcia di Catanzaro, Napoli, Campania, Italy; Reykjavik University, Reykjavik, Hofuoborgarsvaoio, Iceland; Reykjavik University, Reykjavik, Hofuoborgarsvaoio, Iceland

## Abstract

**Background:**

Age-related functional decline emphasizes the need to investigate how tissue composition impacts mobility and independence in older adults. One of the most critical functions associated with mobility disability among older adults is leg muscle strength. The current study investigated the significance of lower leg tissue composition asymmetry on leg muscle strength changes over 5 years among older adults.

**Methods:**

A community-based population from Reykjavik, Iceland (n=3156, mean age=74.9) participated in a 5-year longitudinal study. Tissue composition asymmetry was defined by differences between the right and left sides of non-linear Trimodal Regression Analysis (NTRA) parameters assessed by CT scans at baseline. Among the total of 3156 study participants, 2737 were available for the longitudinal analysis. The maximum strength of the knee extension on the dominant side was measured by using a computerized dynamometer attached to a stable chair at baseline and follow-up assessment. Linear regression analysis was performed with covariates adjustment including age, gender, body mass index, and other lifestyle-related factors.

**Results:**

The results of the study indicate that, after adjusting for covariates, two baseline asymmetry indicators (the amplitude of fat tissue (p< 0.04) and the location of muscle tissue (p< 0.03)) were significantly associated with changes in leg strength over 5 years.

**Conclusion:**

The findings show that higher baseline leg asymmetry indicators, involving both fat and muscle tissue, can predict a greater decline in leg muscle strength during the 5-year follow-up. The composition asymmetry of tissues could serve as an important indicator for predicting mobility disability among older adults.

